# Continuing Professional Development – Radiation Therapy

**DOI:** 10.1002/jmrs.728

**Published:** 2023-09-29

Maximise your CPD by reading the following selected article and answer the five questions. Please remember to self‐claim your CPD and retain your supporting evidence. Answers will be available via the QR code and online at www.asmirt.org/news‐and‐publications/jmrs, as well as published in JMRS – Volume 71, Issue 4 December 2024.

## Radiation Therapy—Original Article

### An evaluation of the use and efficacy of behavioural therapy when treating paediatric patients with radiation therapy

McCoola B, Outhwaite JA, Lathouras M, Pelecanos A, Blyth J, Carter A, Hastings Y, Rattray G, Cheuk R. (2023) *J Med Radiat Sci*. https://doi.org/10.1002/jmrs.705

1. When are behavioural therapy techniques incorporated into paediatric care at the Royal Brisbane and Woman's Hospital (RBWH)?
  - At the request of the family.
  - Only to paediatrics aged under 12 years of age.
  - Only additional play appointments booked.
  - For all radiation therapy appointments.
2. Which of the following behavioural therapy practices are used by Radiation Therapists?
  - Cognitive distraction, desensitisation, behavioural rehearsal, and positive reinforcement.
  - Cognitive distraction and aversion therapy.
  - Contingency management and positive reinforcement.
  - Cognitive distraction, aversion therapy and behavioural rehearsal.
3. Over the 4.5‐year study period, the RBWH delivered 5402 occasions of services to 257 paediatric patients. How many were delivered with general anaesthetic (GA)?
  - 1000 (18.5%)
  - 1361 (25.2%)
  - 1756 (32.5%)
  - 3176 (58.8%)
4. In this study, which of the following treatment or patient variables was associated with anaesthesia use?
  - Male gender.
  - Use of mask for stabilisation.
  - Treatment technique times of more than 15 min.
  - No variables were found to be significant.
5. What was the finding of this study for 3‐ to 8‐year‐olds who used play appointments and were categorised as ‘GA plus Play’ after CT Planning?
  - All 3–8‐year‐olds who were categorised as ‘GA plus play’, stayed as full GA.
  - 10% (3/30) who were categorised as ‘GA plus play’, proceeded to treatment with no GA.
  - 53.3% (16/30) who were categorised as ‘GA plus play’, changed their treatment anaesthetic outcome and ceased GA before or during treatment.
  - 33.3% (10/30) who were categorised as ‘GA plus play’, stayed as full GA.

### Recommended further reading:

1. Jacques A, Udowicz M, Bayliss Y, Jensen K. Thinking differently about the kids: an innovative approach to improve care provided to pediatric patients undergoing external beam radiation therapy. *J Med Imaging Radiat Sci*. 2014 Sep;45(3):269–275. doi: 10.1016/j.jmir.2013.12.009
2. Scott MT, Todd KE, Oakley H, et al. Reducing anesthesia and health care cost through utilization of child life specialists in pediatric radiation oncology. *Int J Radiat Oncol Biol Phys*. 2016 Oct 1;96(2):401–405. doi: 10.1016/j.ijrobp.2016.06.001
3. Ntoukas SM, Ritchie T, Awrey S, et al. Minimizing general anesthetic use in pediatric radiation therapy. *Pract Radiat Oncol*. 2020 May‐Jun;10(3):e159–e165. doi: 10.1016/j.prro.2019.12.001
4. Haeberli S, Grotzer MA, Niggli FK, et al. A psychoeducational intervention reduces the need for anesthesia during radiotherapy for young childhood cancer patients. *Radiat Oncol*. 2008 Jun 4;3:17. doi: 10.1186/1748‐717X‐3‐17

## Answers


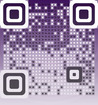


Scan this QR code to find the answers, or visit www.asmirt.org/news‐and‐publications/jmrs

